# Editorial: Impact of plant bioactive compounds on human nutrition and health

**DOI:** 10.3389/fnut.2026.1929598

**Published:** 2026-10-23

**Authors:** Iwona Kowalska, Karolina Wojtunik-Kulesza

**Affiliations:** 1 Department of Phytochemistry, Institute of Soil Science and Plant Cultivation, State Research Institute, Puławy, Poland; 2 Department of Inorganic Chemistry, Medical University of Lublin, Lublin, Poland

**Keywords:** bioactive compounds, disease prevention, human health, human nutrition, secondary metabolites

Epidemiological studies have revealed that health problems are increasingly occurring at younger ages. Considering the phenomenon of population aging, lifestyle-related diseases are a major global health challenge (1). It should be emphasized that many of these disorders are closely associated with nutrition and dietary habits. Growing evidence suggests that diets rich in plant-based food support metabolic health and may reduce the incidence of chronic non-communicable diseases (2). Despite increasing awareness of the impact of diet on our health, we are seeing a rise in the incidence of diet-related diseases and a decline in physical activity (3). One possible way to counteract this phenomenon is to improve dietary habits by including plenty of vegetables, fruit, unprocessed foods, and functional foods tailored to specific needs.

Plants are an inexhaustible source of bioactive compounds, which play a crucial role not only in plant physiology but also in human health. Since ancient times, secondary plant metabolites remain a key resource in modern pharmaceutical research and drug development (4, 5). Scientists around the world are focused on identifying novel bioactive compounds of natural origin with pro-health properties. To date, thousands of compounds have been isolated and identified as promising pro-health molecules, revealing a wide spectrum of biological activities (6). It is extremely important for multidirectional strategy development of drugs for multifactorial diseases (7, 8). Properly functioning organisms need an interdisciplinary approach to ensure the provision of high-quality food containing pro-health components, such as aforementioned secondary plant metabolites. One possible solution is functional food, which is a perfect example of an interdisciplinary approach to food and food technology (9). Products enriched with vegetables, fruits, and herbs are sources not only of essential nutrients but also of bioactive compounds that support proper cellular functions. Increasing evidence supports the inclusion of bioactive-rich plant foods in the diet as an effective strategy. The approach makes it possible to replace unhealthy, high-calorie snacks with health-promoting alternatives that are equally tasty, significantly lower in calories, and provide additional health benefits (10).

Nevertheless, the translation of the promising biological activities of plant-derived compounds into health benefits remains challenging. Their bioavailability, absorption, metabolism, and chemical stability may influence their biological effects, while concentrations used in experimental studies are often higher than those achievable through normal dietary exposure (11, 12). Long-term safety, along with potential interactions with medications or other dietary components, should be carefully considered. Therefore, further research should aim not only to identify bioactive compounds and their mechanisms of action but also to establish physiologically relevant doses, pharmacokinetic profiles, and long-term safety to support their effective and evidence-based application in functional foods and preventive nutrition (13).

The aim of the Research Topic was to highlight the role of plants and natural bioactive compounds in promoting health and disease prevention. The latest advances in the study of plant bioactives and their implications for human nutrition and health were explored, aiming to bridge the gap between food science, clinical research, and public health. The Research Topic helps translate scientific knowledge into practical dietary strategies and health policies.

Given the progress made in identifying compounds, analyzing their activity, and exploring their potential applications, there are unlimited possibilities for using them in a wide range of fields. The pharmaceutical and food industries are the most extensively studied in this regard, as they make extensive use of the benefits offered by nature. We are witnessing a return to natural compounds and their use in the prevention and treatment of many diseases.

Curcumin is being used as a theranostic agent to develop integrated diagnostic platforms for the treatment of complex chronic diseases, with particular emphasis on oncology and neurodegenerative disorders (Yasir et al.). Curcumin's inherent fluorescence, affinity for pathological proteins, and modulation of key molecular pathways underlie its potential as both an imaging probe and a medicinal molecule. A significant number of preclinical studies demonstrates the potential benefits of using curcumin both as a contrast agent and as a substance with anti-cancer or anti-disease effects in Alzheimer's disease. However, this has not been reflected in an equally large number of clinical trials investigating theranostic applications in either of these conditions (14). Future research should focus on providing a more detailed explanation of how the formulation affects therapeutic activity when using biologically active curcumin-based imaging agents, conducting more comprehensive analyses of multimodal theranostic approaches involving curcumin, and systematically investigating combination anti-cancer therapy regimens that incorporate an imaging component in the form of a curcumin preparation. These efforts may facilitate a more comprehensive exploitation of curcumin's theranostic potential and inform the design of clinical trials incorporating imaging techniques (15).

There has been growing interest in using natural bioactive compounds to slow the skin-aging process. Proanthocyanidins have significant potential to improve the structure and function of aging skin (Ye et al.). Leafy green vegetables (e.g., spinach, arugula, lettuce, etc.) are also crucial for healthy aging due to their polyphenol and carotenoid content. Non-thermal processing techniques, such as pulsed electric fields and ultrasound, preserve the antioxidant capacity of bioactive compounds, improve interactions with digestive enzymes, and increase their bioavailability (Ibrahim et al.). Phytoestrogens also play a role in anti-aging and alleviating menopausal symptoms. Experimental data suggest that phytoestrogens strengthen the defense mechanisms of the genitourinary system through their antibacterial, anti-inflammatory, and circulation-enhancing effects (16). In preclinical studies, phytoestrogens have demonstrated protective potential in the context of menopause, acting through the selective activation of ERα, ERβ, and GPER receptors and the modulation of signaling pathways. The results of current clinical trials suggest their usefulness as an alternative to menopausal hormone therapy (MHT, formerly known as HRT) in the treatment of menopausal symptoms. Furthermore, well-designed, large-scale studies are needed to confirm their potential in clinical practice (Li Y. et al.).

The increased incidence of diseases associated with free-radical-induced cellular damage is driving growing demand for naturally derived antioxidants. A new antioxidant peptide (VPNHFNAP) from black soybean [*Glycine max* (L.) Merr.] possesses potent cytoprotective effects and multilevel antioxidant activity. Its dual function suggests potential applications in functional foods and cosmetics (Zhu et al.).

Disease prevention is a key public health measure. Sleep is crucial for human health. Recently, research has been conducted on dietary phenolic compounds and sleep health. Pinaffi-Langley et al. demonstrated that urolithin A improves mitochondrial function, modulates the central clock, and protects against sleep deprivation-induced neuroinflammation.

Food products that reflect the similarities between drugs and food are also important in the transition from a passive approach to health toward proactive prevention. They demonstrate significant medicinal value (e.g., hypoglycemic) and dietary benefits (Li T. et al.). Understanding the potential benefits of dietary fiber consumption is also crucial for developing targeted public health interventions. There is a relationship between its consumption and mortality among adults with metabolic syndrome (Guo et al.). Olive leaf tea (*Olea europaea* L.) also has medicinal benefits, containing antioxidants such as oleuropein, quercetin, hydroxytyrosol, and its derivatives. The biophenols present in olive leaves support healthy intestinal microflora and reduce the likelihood of intestinal disorders (Zafar et al.).

The positive effects of natural bioactive compounds can be easily observed in the Mediterranean diet, which is rich in plant-based foods (17, 18). Adherence to this dietary pattern may help reduce the risk of a wide range of health problems associated with the consumption of highly processed foods.

The findings highlight the growing potential of plant-derived compounds to support human health and reduce the burden of chronic diseases. Future studies should focus on the translation of experimental findings into clinical practice to evaluate their long-term efficacy and safety. This approach will facilitate the development of evidence-based dietary recommendations while promoting health and a good quality of life. Interdisciplinary collaboration among food scientists, pharmacologists, and clinicians will be essential for developing a new generation of drugs and food products with scientifically validated health benefits.

## Future perspectives

Future research should address key challenges in translating plant-derived bioactives into practical applications. Sustainable extraction and processing methods should maximize bioactive recovery while simultaneously reducing the environmental impact. Improved delivery strategies may enhance the bioavailability and stability of poorly absorbed compounds, while personalized nutrition could help account for interindividual differences in metabolism and response. Integrating these approaches may support safer, more effective, and sustainable use of plant-derived bioactives.
